# An alcohol extract prepared from the male flower of *Eucommia ulmoides* Oliv. promotes synoviocyte apoptosis and ameliorates bone destruction in rheumatoid arthritis

**DOI:** 10.1186/s13020-021-00522-2

**Published:** 2021-11-06

**Authors:** Yan Zhang, Jian-Ying Wang, Hao Wang, Xiao-Yun Chen, Lei Zhang, Ying Yuan

**Affiliations:** 1 School of Pharmacy, Shanghai University of Traditional Chinese Medicine, 1200 Cailun Road, Pudong District, Shanghai, 201203 China; 2 Shanghai Innovation Center of TCM Health Service, Shanghai University of Traditional Chinese Medicine, 1200 Cailun Road, Pudong District, Shanghai, 201203 China; 3 Shanghai Key Laboratory of Formulated Chinese Medicines, Shanghai University of Traditional Chinese Medicine, 1200 Cailun Road, Pudong District, Shanghai, 201203 China; 4 Rheumatoid Department, Shanghai Longhua Hospital Affiliated to Shanghai University of TCM, No. 725 South Wanpin Road, Xuhui District, Shanghai, 200232 China

**Keywords:** Rheumatoid arthritis, *Eucommia ulmoides* Oliv., CIA rat, Bone metabolism, NF-κB signaling pathway

## Abstract

**Background:**

Rheumatoid arthritis (RA) is a chronic systemic autoimmune disease dominated by synovial hyperplasia and bone destruction. The male flower of *Eucommia ulmoides* Oliv. (EF) has been shown to exert effects on the inflammation caused by RA. However, how EF affects synoviocyte apoptosis and bone destruction on RA have not been investigated thoroughly. The effects of EF on apoptosis of human fibroblast-like synoviocytes-rheumatoid arthritis (HFLS-RA) cells, osteoclast differentiation of RAW264.7 cells, and bone destruction in a collagen-induced arthritis (CIA) model in rats were explored.

**Methods:**

First, the main components of EF were identified by high-performance liquid chromatography. In vitro, we investigated the anti-proliferative and pro-apoptotic effects of EF on HFLS-RA cells by immunofluorescence assays, flow cytometry, real-time reverse transcription-quantitative polymerase chain reaction (RT-qPCR), and western blotting; we also investigated how EF influenced the differentiation of RAW264.7 cells into osteoclasts. In vivo, we used a rat model of CIA to investigate the effects of EF on anti-arthritis activity, toe swelling, Arthritis Score, serum levels of metabolic bone factors, and pathologic conditions. Micro-computed tomography was used to scan ankle joints. mRNA and protein expression of factors related to the nuclear factor-kappa B (NF-κB) pathway were determined by RT-qPCR and western blotting, respectively.

**Results:**

EF inhibited synoviocyte proliferation and promoted apoptosis in a dose-dependent manner. EF inhibited osteoclast differentiation by inhibiting activation of the NF-κB pathway. EF reduced articular inflammation in CIA rats, inhibited the expression of pro-angiogenic factors, and delayed the destruction of articular cartilage and bone. Our data indicated that EF acted via a mechanism related to bone metabolism induced by the NF-κB pathway.

**Conclusions:**

EF exerts a potential therapeutic effect upon RA. Our research will help to elucidate the potential pharmacologic mechanisms associated with the beneficial effects of EF, and provide an experimental basis for EF application in clinical treatments.

**Graphical Abstract:**

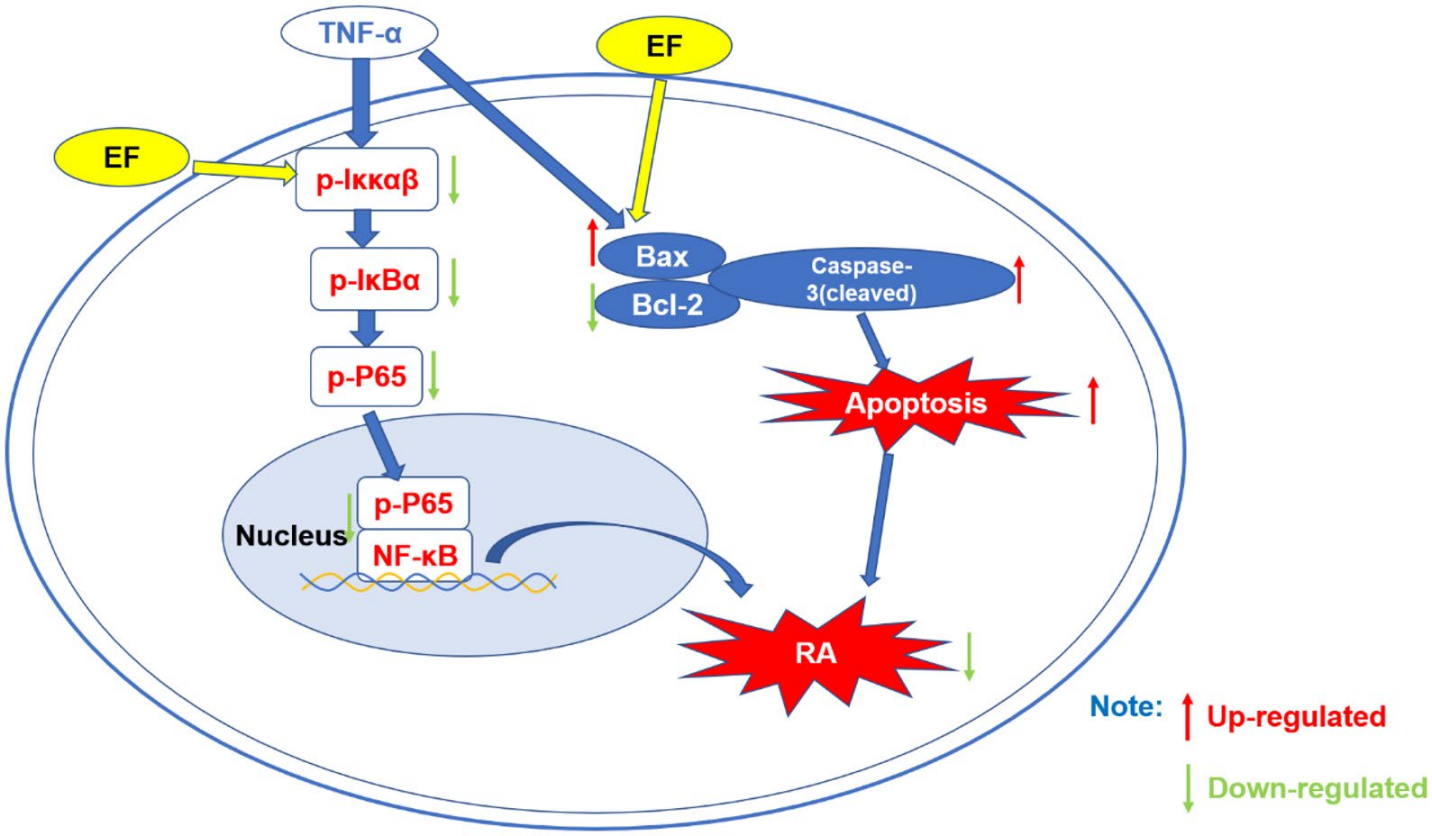

## Background

Rheumatoid arthritis (RA) is an autoimmune disease dominated by chronic multiarticular inflammation and bone destruction. The main pathologic features of RA are the proliferation of synovial cells, pannus formation, destruction of cartilage and bone, and joint deformity. These changes cause a great deal of inconvenience to patients affected by RA, and create a significant burden with regard to daily life and work activities.

The World Health Organization lists RA as one of the most troublesome global diseases [1]. Diagnostic methods and treatments have improved significantly over recent years. Drugs for RA are mainly disease-relieving anti-rheumatic drugs [e.g., methotrexate (MTX)], non-steroidal anti-inflammatory drugs, glucocorticoids (e.g., dexamethasone), and other biologic agents [2]. Even if synovial inflammation has been controlled during the late stages of RA, bone destruction can cause irreversible damage to joints. Therefore, inhibiting the early proliferation of synovial cells could reduce the subsequent erosion of cartilage and bone. Traditional Chinese medicine (TCM) has achieved curative effects in RA treatment. Previous studies have found that three extracts from *Eucommia ulmoides* Oliv. (EU) can significantly inhibit bone destruction, synovial inflammation, and systemic inflammation [1].

The nuclear factor-kappa B (NF-κB) pathway has important roles in various immune diseases. If stimulated by interleukin (IL)-1 and tumor necrosis factor (TNF)-α, cells activate IκB kinase (Iκκs); this kinase phosphorylates, ubiquitinates, and then degrades the IκB protein. This action leads to the dissociation of IκB from NF-κB in the cytoplasm. Once transferred to the nucleus, NF-κB regulates the transcription of a range of target genes, promotes angiogenesis and the expression of cytokines such as IL-1β and vascular endothelial growth factor (VEGF) and, thus, exacerbates attacks on the bone and cartilage in RA [3]. Therefore, inhibition of the NF-κB pathway could control the bone destruction observed in RA.

There is increasing evidence that Chinese herbal medicines are valuable resources for the treatment of some intractable diseases [4, 5]. The bark and leaves of EU are used widely in TCM clinics. The active ingredients of EU can reduce blood pressure, improve immune function, and can exhibit anti-aging, anti-inflammatory, and anti-tumor effects. EU is one of the most commonly prescribed TCM ingredients used for the clinical treatment of RA [6, 7]. Previously, we found that an alcohol extract prepared from the bark of *Eucommia ulmoides* Oliv. (EB) could inhibit the proliferation of fibroblast-like synoviocytes in human RA in vitro and promote their apoptosis. In vivo, the swelling and Arthritis Score of a model of collagen-induced rheumatoid arthritis (CIA) in rats were reduced upon EB treatment; pathology further suggested that infiltration of inflammatory cells into the synovium was improved and pannus formation was alleviated [8]. However, EB grows slowly and resources are incredibly scarce, so there is an urgent need to identify alternatives.

The male flower of *Eucommia ulmoides* Oliv. (EF) is relatively rich in terms of resources. EF has been shown to exert anti-inflammatory, analgesic, antibacterial, and other pharmacologic effects, including immunoregulation [9]. EF is often used for daily healthcare, and the prospects are good for developing and utilizing EF in a wide range of clinical applications [10]. Previously, we preliminarily discussed the effects of EB, leaf of *Eucommia ulmoides* Oliv (EL), and EF on improving inflammation in rats suffering from CIA (hereafter termed “CIA rats”). EF and EB have similar functions. We found that EF can reduce the expression of TNF-α and nitric oxide (NO) in synoviocytes and improve articular inflammation in CIA rats in vivo [11]. On that basis, we wished to: (i) explore how EF promotes the apoptosis of synoviocytes and inhibits bone destruction; (ii) explore whether the effect of EF on apoptosis of synoviocytes and bone destruction on CIA rats was related to activation of the NF-κB pathway; (iii) provide an experimental basis for future application of EF. Currently, few studies have focused on RA treatment using EF.

Here, we investigated the effect of EF on the: (i) proliferation, migration, and apoptosis of human fibroblast-like synoviocytes in rheumatoid arthritis (HFLS-RA); (ii) osteoclast differentiation in RAW264.7 cells; (iii) bone destruction via the NF-κB pathway in a rat model of CIA. Our goal was to provide an experimental basis for the rational application of EF in clinical RA treatments, and interpret its pharmacologic mechanism of action.

## Materials and methods

### Main reagents and antibodies

Dulbecco’s modified Eagle medium (DMEM) was purchased from Gibco (catalog number, 11995065; Grand Island, NY, USA). Human TNF-α was obtained from Peprotech (315-01 A; Rocky Hill, NJ, USA). Anti-NF-κB-phospho-p65, anti-NF-κB-p65, anti-phospho-Iκκαβ, anti-Iκκα, anti-phospho-IκBα, anti-IκBα, anti-cleaved cysteinyl aspartate specific proteinase (caspase) 3, anti-bcl-2-associated X protein (Bax), and anti-B-cell lymphoma (bcl)-2 receptor antibodies, along with protease inhibitors, were purchased from Cell Signaling Technology (3033, 8242, 2697, 11930, 2859, 4814, 9644, 5023, and 15071, respectively; Beverly, MA, USA). Bovine type II collagen (CII) and complete Freund’s adjuvant (CFA) were provided by Chondrex (20021, and 7001, respectively; Seattle, WA, USA). Rat enzyme-linked immunoassay (ELISA) kits (C-terminal telopeptide I [CTX-1], Cross-linked carboxy-terminal telopeptide of type I collagen [ICTP], Propeptide of type I procollagen [PINP], and Bone galprotein [BGP] were obtained from Kenuodi Biological Technology (TF129, TF176, TF143, TF181; Kenuodi, China). EZ-press RNA Purification Kit, 4× Reverse Transcription Master Mix, and 2× SYBR Green qPCR Master Mix, were purchased from EZBioscience (B0004DP, A0010CGQ, and A0012-R2, respectively; Roseville, CA, USA). MTX was purchased from Shanghai Shang Yao Xinyi Pharmaceutical Factory (2.5 mg/tablet, 16 tablets/bottle; batch number, 036170604).

### Ethanol extracts of plant material

EF was purchased from Shanghai Kangqiao Chinese Medicine Tablets (Shanghai, China) and was identified as the dried male flower of EU by Wu Jin-Rong (Associate Professor, Department of Pharmacognosy, School of Chinese Materia Medica, Shanghai University of Chinese Medicine).

EF was extracted twice using 70% ethanol (1:8, w/v for 1 h), filtered, and vacuum-dried before experimentation. The concentration of the extract was 1 g/mL (equivalent to the original herb) [11]. Based on preliminary experiments conducted by our research team, the effect was obvious if the drug concentration was 1 g/mL. Therefore, in in vivo experiments, the concentration of the high dose was set at 1 g/mL and the low dose was set at 0.5 g/mL.

### Quality control of EF

To investigate the main constituents of the ethanol extracts of EF qualitatively, we undertook high-performance liquid chromatography (HPLC). We undertook HPLC of the chemical constituents in the ethanol extract of the male flower of EU using a 1260 system from Agilent Technologies (Santa Clara, CA, USA). Mobile phase acetonitrile (phase A) was 0.1% phosphoric acid aqueous solution (phase B). The order was: 0 min, 4% A; 5 min, 10% A; 35 min, 23% A; 45 min, 60% A; 50 min, 90% A; 60 min, 90% A; 60.01–80 min, 4% A. We used a Cosmosil 5C18 AR-II column (Cosmosil, Japan). The absorbance wavelengths were 238 nm and 194 nm. The elution speed was 1.0 mL/min and the temperature was 25 °C. As stated above, the concentration of the extract was 1 g/mL. The extract was transferred to a 100-mL volumetric flask, and made up to 100 mL. The concentration of the reference standards was formulated as follows: pinoresinol diglucoside (0.0816 mg/mL), geniposidic acid (0.0368 mg/mL), chlorogenic acid (0.038 mg/mL), geniposide (0.0396 mg/mL), genipin (0.0408 mg/mL), and quercetin (0.0204 mg/mL). The content of these components could be calculated using the external standard two point method according to the standard concentration.

### Culture and viability assays for HFLS-RA cells

HFLS-RA cells were purchased from Huatuo Biotechnology (Guangzhou, China). They were cultured in DMEM supplemented with 10% fetal bovine serum (FBS) and 1% penicillin–streptomycin solution (hereafter referred to as “standard growth medium”) in a humidified atmosphere of 5% CO_2_/95% air at 37 °C (referred to hereafter as “standard culture conditions”). The Cell Counting Kit (CCK)-8 assay (Dojindo, Kunamoto, Japan) was used in accordance with manufacturer instructions to determine cell viability. HFLS-RA cells were pre-incubated for 24 or 48 h with EF (0, 25, 50, 100, 200, 400, 800, 1600 µg/mL) in 96-well plates. Then, 10% CCK8 medium was added to the 96-well plates and a microplate reader (SpectraMax iD5, Molecular Devices, USA) was used to measure the absorbance at 450 nm. Cell viability was expressed as a proportion (%) compared with that in the control group.

### Animals

Thirty Wistar rats (120 ± 10 g) were purchased from Beijing CRL Laboratory Animals (animal certificate number, SCXK (Beijing) 2016-0011) in Beijing, China, to generate the CIA model. Animal procedures were undertaken according to the ethical guidelines set by the Laboratory Animal Welfare and Animal Experimental Ethics Committee of Shanghai University of Traditional Chinese Medicine (approval number: PZSHUTCM18122823) in Shanghai, China.

### Cell proliferation and the CCK-8 assay

HFLS-RA cells were inoculated in 96-well plates. Cells were pre-incubated for 24 h with EF (0, 25, 50, 100, 200, 400, 800, 1600, 2000 µg/mL) with or without TNF-α (10 ng/mL) for 24 h. Following stimulation, 10% CCK8 medium was added to the 96-well plates. A microplate reader was used to measure the absorbance at 450 nm. Cell proliferation was expressed as a proportion (%) compared with that in the control group.

### Cell colony-forming assay

Collagen from rat tails was diluted to 3% with phosphate-buffered saline and added to a six-well plate. HFLS-RA cells were cultured in serum-free DMEM for 24 h in six-well plates (in triplicate). After starvation, cells were left untreated (negative control) or supplemented with EF (0, 100, 200, 400, 800, 1600 µg/mL). Cells were treated for 2 days and then cultured for 7 days. Following culture, cells were fixed with 4% pre-cooled paraformaldehyde and stained with 0.5% crystal violet. The latter was eluted with 70% ethanol, and the eluent was transferred to a 96-well culture plate. Then, the absorbance was read at 595 nm. Cell viability was expressed as a proportion (%) compared with that in the control group.

### Cell proliferation and the 5-ethynyl-2′-deoxyuridine (EdU) assay

HFLS-RA cells were cultured in serum-free DMEM for 24 h. Next, cells were left untreated (negative control) or supplemented with EF (400, 800, 1600 µg/mL) and TNF-α (10 ng/mL) for 24 h. Cell-proliferation assays were undertaken using a BeyoClick™ EdU Cell Proliferation Kit with Alexa Fluor 594 (Beyotime Institute of Biotechnology, Shanghai, China) in accordance with manufacturer instructions.

### Tests to measure the migration and invasion of cells

The chemotaxis tool plugin “Ibidi” (ImageJ, National Institutes of Health, Bethesda, MD, USA) was placed in a 24-well culture plate. Then, HFLS-RA cells were inoculated into the plugin and left untreated (negative control) or supplemented with EF (400, 800, 1600 µg/mL) and TNF-α (10 ng/mL) for 24 h. Scratch areas were photographed with an inverted microscope (Leica, Germany). Cells were also implanted in Transwell™ chambers and left untreated (negative control) or supplemented with EF (400, 800, 1600 µg/mL) and TNF-α (10 ng/mL) for 24 h. Following treatment, cells were fixed with 4% paraformaldehyde and stained with 0.1% crystal violet solution. Then, cells were placed onto a glass slide, and five fields from each group were selected for photography under an inverted microscope. Cells were eluted with 33% acetic acid in 96-well plates and the absorbance read at 570 nm. Cell viability was expressed as a proportion (%) compared with that in the control group.

### Flow cytometry to measure apoptosis

HFLS-RA cells were pre-incubated for 24 h with EF (400, 800, 1600 µg/mL) with or without TNF-α (10 ng/mL). As recommended by the manufacturers of the Annexin V-fluorescein isothiocyanate (FITC)/propidium iodide (PI) apoptosis detection kit (556,547, Becton Dickinson, USA), cells were resuspended with binding buffer, and mixed with 5 µL of Annexin V-FITC and PI. Apoptosis was detected by flow cytometry (Becton Dickinson).

### Measurement of mRNA expression of proinflammatory factors in HFLS-RA cells by real-time reverse transcription-quantitative polymerase chain reaction (RT-qPCR)

HFLS-RA cells were cultured in six-well plates and treated with TNF-α (10 ng/mL) and EF (400, 800, 1600 µg/mL) for 24 h. RNA was extracted with EZ-Press RNA Purification Kit (EZBioscience). Complementary-DNA was synthesized with 4× Reverse Transcription Master Mix (EZBioscience). Real-time RT-qPCR was employed to quantify gene expression. RT-qPCR was undertaken using 2× SYBR Green qPCR Master Mix on an ABI Prism 7500 qPCR system (Thermo Fisher Scientific, Waltham, MA, USA) with initial denaturation at 95 °C for 5 min, followed by 40 cycles of 95 °C for 10 s, 60 °C for 30 s, and a final extension at 72 °C for 90 s. The primers used for RT-qPCR are shown in Table 1. Data were normalized to the expression of glyceraldehyde 3-phosphate dehydrogenase (GAPDH) using the 2^−ΔΔCT^ method [1]. All experiments were repeated thrice.

**Table 1:** Sequences of the primers used for RT-qPCR to determine the mRNA expression of proinflammatory factors in HFLS-RA cells

Gene	5′–3′	Sequence
*GAPDH*	Forward	CATGAGAAGTATGACAACAGCCT
	Reverse	AGTCCTTCCACGATACCAAAGT
*IL-1β*	Forward	TTCGACACATGGGATAACGAGG
	Reverse	TTTTTGCTGTGAGTCCCGGAG
*IL-6*	Forward	ACTCACCTCTTCAGAACGAATTG
	Reverse	CCATCTTTGGAAGGTTCAGGTTG
*MMP-9*	Forward	TGTACCGCTATGGTTACACTCG
	Reverse	GGCAGGGACAGTTGCTTCT
*VEGF*	Forward	AGGGCAGAATCATCACGAAGT
	Reverse	AGGGTCTCGATTGGATGGCA
*Caspase3* *(cleaved)*	Forward	TTACAGTGAACACCTCTACCAATGCCCCA
	Reverse	TCCGTGAAAACACTAATCACCTTGCCCCA
*Bax*	Forward	GGGAGACACCTGAGCTGACC
	Reverse	GGACTCCAGCCACAAAGATGG
*Bcl-2*	Forward	GAACTGGGGGAGGATTGTGGCC
	Reverse	TCGACGTTTTGCCTGAAGACTGTTAA

### Expression of the proteins related to the NF-κB pathway in HFLS-RA cells using western blotting

HFLS-RA cells were cultured in six-well plates for 12 h and then treated with TNF-α (10 ng/mL) and EF (400, 800, 1600 µg/mL) for 24 h. RIPA lysate (including a protease inhibitor and phosphatase inhibitor) was used to lyse cells. Protein concentrations were determined with a bicinchoninic acid protein assay kit (Meilunbio, Dalian, China). Then, equal concentrations of each protein lysate from each group were mixed with loading buffer, separated by sodium dodecyl sulfate–polyacrylamide gel electrophoresis (EpiZyme, Beijing, China), and then electro-transferred to nitrocellulose membranes. The latter were blocked with 5% skimmed milk powder and incubated overnight with primary antibody at 4 °C. The following morning, the nitrocellulose membranes were incubated with secondary antibody at room temperature, and immunoreactive bands were detected by enhanced chemiluminescence. The gray value of the protein bands was analyzed. The ratio of the target protein to GAPDH was used to evaluate the expression of each protein. All experiments were repeated thrice.

### Cell-viability assays for RAW264.7 cells

RAW264.7 cells were purchased from Huatuo Biotechnology. The CCK-8 assay was used (in accordance with manufacturer instructions) to determine cell viability. RAW264.7 cells were pre-incubated for 24 h with EF (0, 50, 100, 200, 400, 800, 1600, 2000 µg/mL) in 96-well plates. Then, 10% CCK8 medium was added to the 96-well plates and a microplate reader was used to measure the absorbance at 450 nm. Cell viability was expressed as a proportion (%) compared with that in the control group.

### NO release in RAW264.7 cells

RAW264.7 cells were pre-incubated for 24 h with EF (0, 50, 100, 200, 400, 800, 1600, 2000 µg/mL) in 96-well plates. Then, the cell supernatant was aspirated and Griess medium was added to the 96-well plates. A microplate reader was used to measure the absorbance at 540 nm. NO release was expressed as a proportion (%) of that in the control group.

### Tartrate-resistant acid phosphatase (TRAP) staining

RAW264.7 cells were inoculated in 24-well plates. Next, 10% FBS + 1% penicillin–streptomycin + macrophage colony-stimulating factor (M-CSF; 50 ng/mL) + receptor activator of nuclear factor kappa-Β ligand (RANKL; 50 ng/mL) were used as the inducer in each group. Cells were pre-incubated with EF (400, 800, 1600 µg/mL) and induced for 7 days, and the solution was changed every other day. Cells were stained with a TRAP staining kit (387; Sigma-Aldrich, Saint Louis, MO, USA) and observed under a microscope (Leica). Red cells with > 3 nuclei were assigned as osteoclasts.

### Expression of the proteins related to the NF-κB pathway in RAW264.7 cells using western blotting

RAW264.7 cells were inoculated in 24-well plates. Next, 10% FBS + 1% penicillin–streptomycin + M-CSF (50 ng/mL) + RANKL (50 ng/mL) were used as the inducer in each group. Cells were pre-incubated with EF (400, 800, 1600 µg/mL) and induced for 7 days, and the solution was changed every other day. Then, cells were treated with TNF-α (10 ng/mL) for 24 h. Proteins were separated and subject to western blotting as described above to determine the expression of proteins related to the NF-κB pathway. All experiments were repeated thrice.

### CIA in rats

The method for generating the CIA model in rats has been described [1]. In brief, ~ 3 mg/mL of bovine CII in 0.1 M acetic acid was emulsified with an equal volume of complete Freund’s adjuvant (CFA) to create a stable CII/CFA emulsion (10 mg/mL). With the exception of the control group (six rats), all rats were injected (i.d.) with the CII/CFA emulsion in the back (0.1 mL) and tail (0.1 mL); the day of administration was referred to as “day-0”. On day-7, CIA rats received 0.1 mL of a CII/CFA booster *via* tail injection (i.d.).

**Fig. 1:**
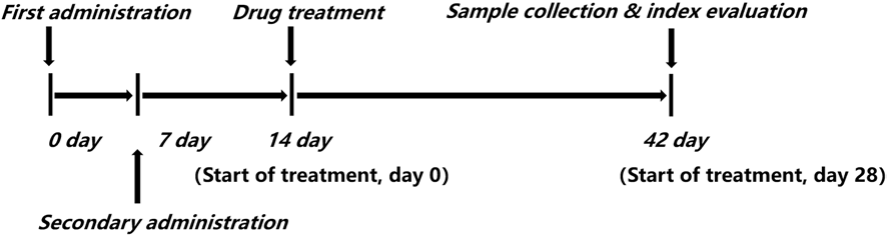
Flowchart for the animal experiment

After 14 days of modeling, the degree of posterior plantar redness and swelling of CIA rats was significantly different from that of the blank control group according to measurement of the plantar volume and Arthritis Score (P < 0.05). These observations were in accordance with the standard required for an arthritis model, which suggested that modeling had been successful [1, 11]. Then, CIA rats were divided randomly into four groups of six. The control group and model group were given 0.1 mL/100 g bodyweight of distilled water; the other groups were given EF at 2 g kg^−1 ^day^−1^ (EF-L) or 4 g kg^−1^ day^−1^(EF-H). The positive control group was given MTX at 0.3 mg kg^−1^ day^−1^ [1]. Dosing was carried out once daily for 4 weeks. At the beginning of day-0, we measured the foot volume in each rat using a plantar volume meter; we also determined the Arthritis Score every 7 days until the end of the experiment (Fig. 1).

### Micro-computed tomography (CT)

On day-42, rats were killed and the right ankle joints fixed in 4% paraformaldehyde. Next, we used a high-resolution micro‐CT system (Skyscan 1176; Bruker, Billerica, MA, USA) to determine bone erosion in the distal ankle joint. Then, we reconstructed a three‐dimensional (3D) structure of the hind paws using Mimics 18.0 (Materialise, Leuven, Belgium). We analyzed several parameters of bone morphometry: percent bone mineral density (BMD), bone volume/tissue volume (BV/TV), mean trabecular number (Tb.N), and mean trabecular separation (Tb.Sp).

### Histopathology

Rats were killed on day-42. The left ankle joints from each rat were taken and fixed in 4% paraformaldehyde. Fixed joints were decalcified with 10% ethylenediamine tetra acetic acid, paraffin-embedded, and then cut into 4-mm slices with a microtome (Leica Microsystems, Wetzlar, Germany). Knee sections prepared from each group were stained with hematoxylin and eosin (H&E). Pathologic improvement in ankle joints was evaluated by examining tissue sections using H&E staining. Synovial inflammation, angiogenesis, and bone destruction was examined in the hind ankle joints and scored under a microscope. The scoring criteria have been reported [1]: 0 points = no infiltration by inflammatory cells, pannus formation, or bone damage; 1 point = infiltration of inflammatory cells or mild edema, and a small amount of pannus formation; 2 points = moderate infiltration of inflammatory cells with pannus formation and moderate bone damage; 3 points = severe infiltration of inflammatory cells with moderate stenosis in the articular cavity; 4 points = pannus formation, and severe bone damage.

### Staining with Safranin O/Fast Green

Histology sections were prepared from the knees of all rats and then stained with the Safranin O/Fast Green Staining Kit (Solarbio, Beijing, China). Pathologic improvements in the ankle joint were evaluated.

### ELISA

Rats were killed on day-42. Blood was taken from the abdominal aorta. Commercial ELISA kits were used to measure the serum levels of IL-1β and TNF-α in accordance with manufacturer instructions (SpectraMax iD5, Molecular Devices, USA). The absorbance was quantified at 450 nm using a microplate reader. Serum concentrations of each factor were calculated by reference to a standard curve.

### Measurement of mRNA expression of proinflammatory factors in a rat model of CIA

Rats were killed on day-42. The spleen and joints were removed from rats in each group. RNA was extracted with the EZ-press RNA Purification Kit. Complementary-DNA was synthesized by 4× Reverse Transcription Master Mix. The primers used for RT-qPCR are shown in Table 2. Data were normalized to GAPDH expression using the 2^−ΔΔCT^ method [1]. All experiments were repeated thrice.

**Table 2:** Sequences of the primers used in RT-qPCR to determine the mRNA expression of proinflammatory factors in a rat model of CIA

Gene	5′–3′	Sequence
*GAPDH*	Forward	CCACCCATGGCAAATTCCATGGCA
	Reverse	TCTAGACGGCAGGTCAGGTCCACC
*TNF-α*	Forward	GGAAAGCATGATCCGAGATG
	Reverse	CGAGCAGGAATGAGAAGAGG
*TRAF-6*	Forward	TCTCGGAGTGCTGCGTGTATAGG
	Reverse	GTCGCTTAGAGACTGGCTGGAC
*IL-1β*	Forward	CTCACAGCATCTCGACAAGAG
	Reverse	CACACTAGCAGGTCGTCATCATCC
*IL-17*	Forward	GCCGAGGCCAATAACTTTCT
	Reverse	GAGTCCAGGGTGAAGTGGAA
*CTSK*	Forward	TAGCACCCTTAGTCTTCCGC
	Reverse	CTTGAACACCCACATCCTGC
*NFATc-1*	Forward	GGAGAGTCCGAGAATCGAGAT
	Reverse	TTGCAGCTAGGAAGTACGTCT
*TRAP*	Forward	TGGGTGACCTGGGATGGATT
	Reverse	AGCCACAAATCTCAGGGTGG
*c-Fos*	Forward	TTTCAACGCCGACTACGAGG
	Reverse	GCGCAAAAGTCCTGTGTGTT
*RANKL*	Forward	AGGCTGGGCCAAGATCTCTA
	Reverse	GATAGTCCGCAGGTACGCTC
*VEGF*	Forward	GGCAGCTTGAGTTAAACGAAC
	Reverse	TGGTGACATGGTTAATCGGTC
*HIF-1*	Forward	GGAAATGCTGGCTCC CTAT
	Reverse	CTGTAACTGGGTCTGCTGGA
*TIMP-1*	Forward	CGAGACCACCTTATACCAGCG
	Reverse	ATGACTGGGGTGTAGGCGTA
*OPG*	Forward	GGCAGGGCATACTTCC TGTT
	Reverse	GCCACTTGTTCATTGTGGTCC

### Expression of proteins related to the NF-κB pathway in a rat model of CIA using western blotting

Rats in each group were killed on day-42. Taking each group as a unit, 20 mg of pooled joint material was ground down, and protein was extracted on ice with RIPA lysate (including a protease inhibitor and phosphatase inhibitor). Proteins were extracted and subject to western blotting as described above to ascertain expression of the proteins related to the NF-κB pathway.

### Statistical analyses

Data were analyzed with SPSS 26.0 (IBM, Armonk, NY, USA). Data are the mean ± standard error of the mean. The significance of differences between groups was evaluated using Student’s *t*-test, p < 0.05 was considered significant.

## Results

### Chemical profiles of the ethanol extract of the male flower of EU

**Fig. 2:**
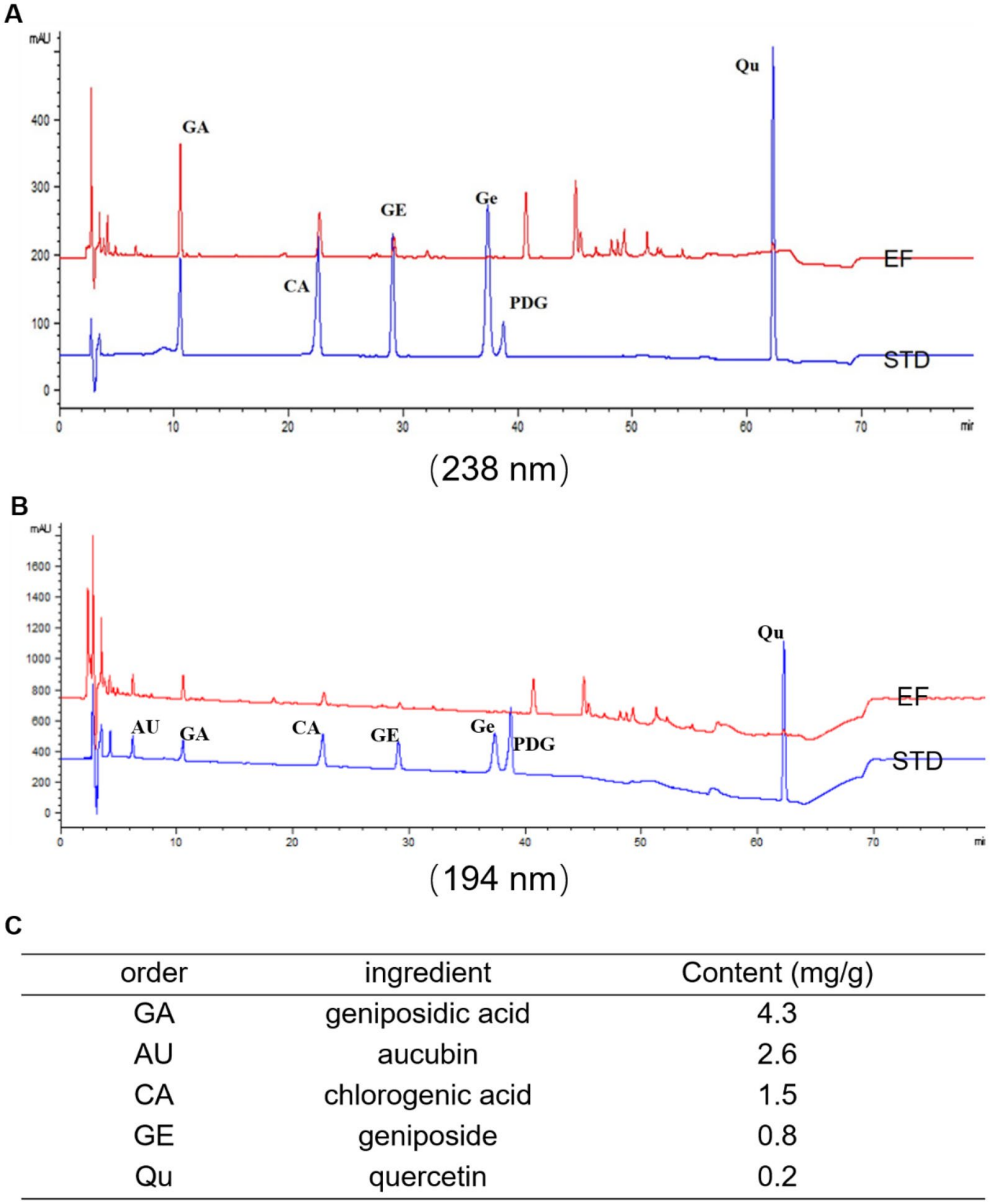
**A** HPLC chromatograms of EF test solution detected at 238 nm. **B** HPLC chromatograms of EF test solution detected at 194 nm. **C** HPLC chromatograms of standard solution and EF test solution detected at 238 nm (GA, CA, GE, Qu) and 194 nm (AU). *GA* geniposidic acid, *CA* chlorogenic acid, *GE* geniposide, *Ge* genipin, *PDG* pinoresinol diglucoside, *Qu* quercetin, *AU* aucubin

HPLC chromatograms of the chemical constituents in the ethanol extract of the male flower of EU are shown in Fig. 2. The main constituent peaks were identified as belonging to geniposidic acid (4.3 mg/g), aucubin (2.6 mg/g), chlorogenic acid (1.5 mg/g), geniposide (0.8 mg/g), and quercetin (0.2 mg/g).

### EF inhibited the proliferation of TNF-α stimulated HFLS-RA cells

**Fig. 3:**
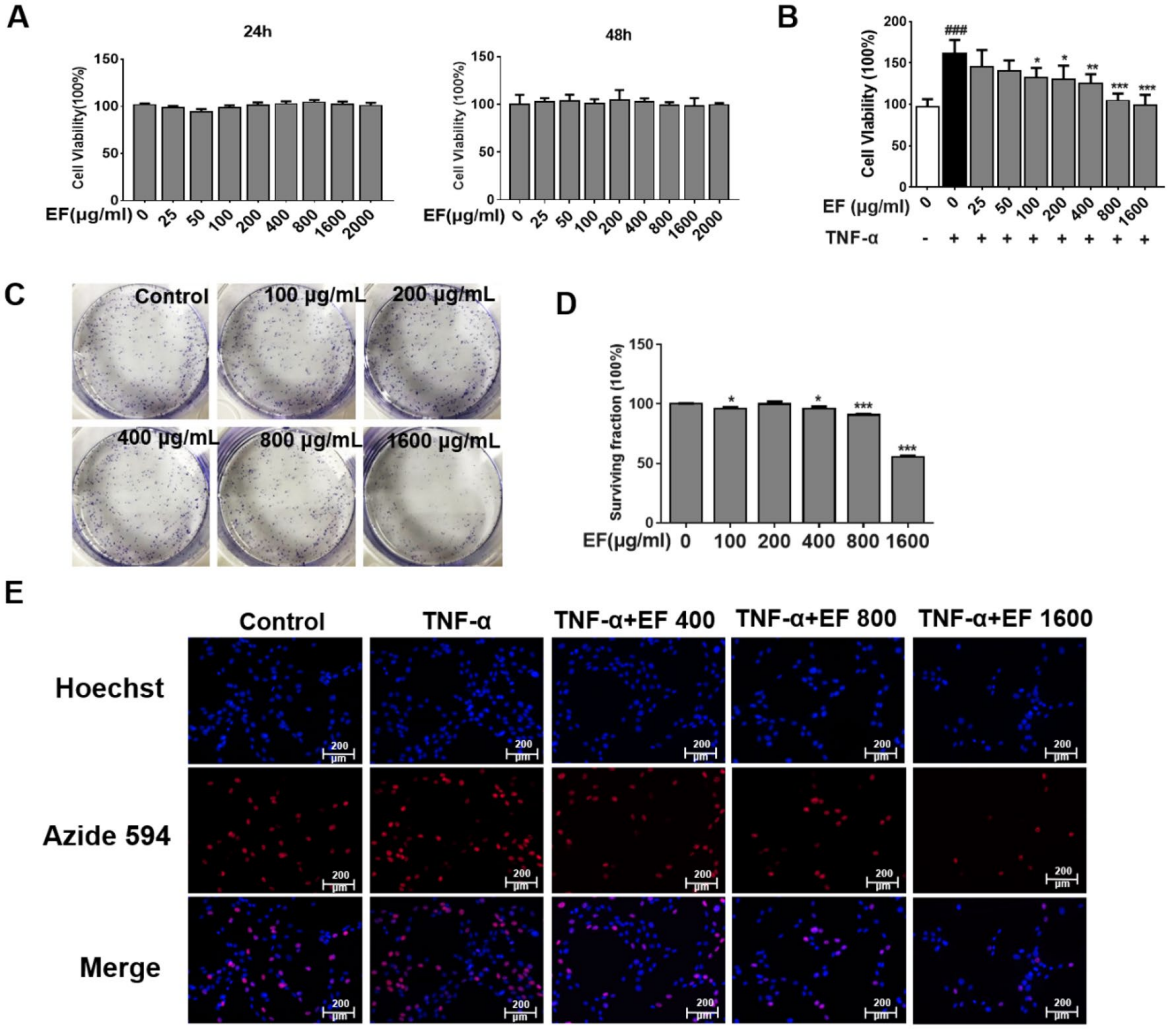
Effects of EF on the proliferation of HFLS-RA cells with and without TNF-α stimulation. **A** Toxicity of EF in cultured cells was tested at 24 and 48 h. **B** Anti-proliferative effects of EF against TNF-α-stimulated HFLS-RA cells exhibited a concentration-dependent relationship. HFLS-RA cells were incubated with TNF-α (10 ng/mL) and EF (25, 50, 100, 200, 400, 800, 1600 µg/mL) for 24 h. The CCK-8 assay was employed to determine the extent to which cell proliferation was inhibited (%). Data are the mean ± SD (n = 4), *p < 0.05, **p < 0.01, ***p < 0.001, vs. TNF-α cells (HFLS-RA cells treated by TNF-α alone). ^#^p < 0.05, ^##^p < 0.01, ^###^p < 0.001, vs. control cells. **C** A cell-colony assay was used to investigate the effect of EF on the proliferation of HFLS-RA cells. **D** Data are the mean ± SD (n = 4), *p < 0.05, **p < 0.01, ***p < 0.001, vs. control cells. **E** HFLS-RA cells were seeded onto glass coverslips, serum-starved, and then stimulated for 24 h with TNF-α (10 ng/mL) or EF (400, 800, 1600 µg/mL). Then, cells were stained with the EdU Cell Proliferation Kit. Hoechst (blue) and Azide594 (red) were visualized by fluorescence microscopy (×200)

The influence of EF on the viability of HFLS-RA cells was investigated with the CCK-8 assay (Fig. 3A). EF was not cytotoxic at 24 or 48 h. EF (400, 800, 1600 µg/mL) could significantly inhibit the proliferation of TNF-α-stimulated HFLS-RA cells (p < 0.05, p < 0.01, p < 0.001) (Fig. 3B). EF (400, 800, 1600 µg/mL) exhibited the best ability to inhibit proliferation (p < 0.05, p < 0.001) (Fig. 3C and D). Therefore, we chose these concentrations of EF (400, 800, 1600 µg/mL) for subsequent experiments. EdU-incorporation assays (Fig. 3E) showed that EF treatment (800, 1600 µg/mL) blocked TNF-α-induced proliferation of HFLS-RA cells significantly. Moreover, the anti-proliferative effect of EF occurred in a concentration-dependent manner.

### EF inhibited the migration and invasion of TNF-α stimulated HFLS-RA cells

**Fig. 4:**
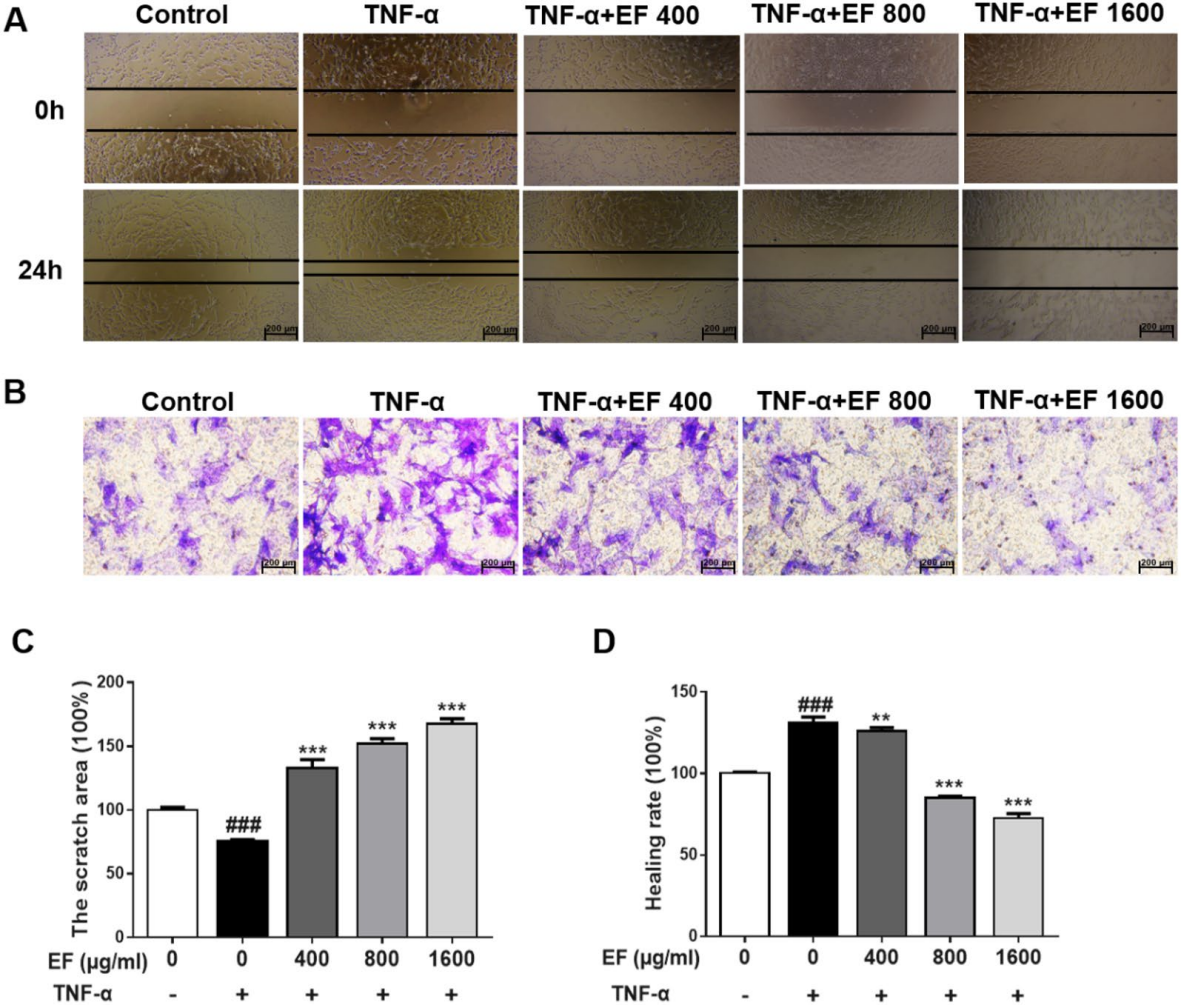
Effects of EF on the migration and invasion of TNF-α-stimulated HFLS-RA cells. HFLS-RA cells were incubated with EF (400, 800, 1600 µg/mL) and TNF-α (10 ng/mL) for 24 h. **A**, **C** Effect of EF on HFLS-RA cells (×100) with regard to wound migration. Cell mobility was calculated and photographed. **B**, **D** An invasion assay was carried out in a chamber using a Matrigel™ basement membrane matrix and invading cells were counted to quantify chemotaxis. Images show the invasion of HFLS-RA cells following EF treatment. Data are the mean ± SD (n = 4), *p < 0.05, **p < 0.01, ***p < 0.001, vs. TNF-α cells (HFLS-RA cells treated by TNF-α alone). ^#^p < 0.05, ^##^p < 0.01, ^###^p < 0.001, vs. control cells

Next, we evaluated the effects of EF on the migration and invasion of HFLS-RA cells (Fig. 4A, C). The scratch area in the EF group was significantly larger than that in the TNF-α group (p < 0.001), which indicated that EF administration inhibited cell migration. We evaluated the effect of EF on the invasion of cells (Fig. 4B, D): EF inhibited cell invasion significantly (p < 0.01, p < 0.001).

### EF induced the apoptosis of TNF-α-stimulated HFLS-RA cells via the NF-κB pathway

**Fig. 5:**
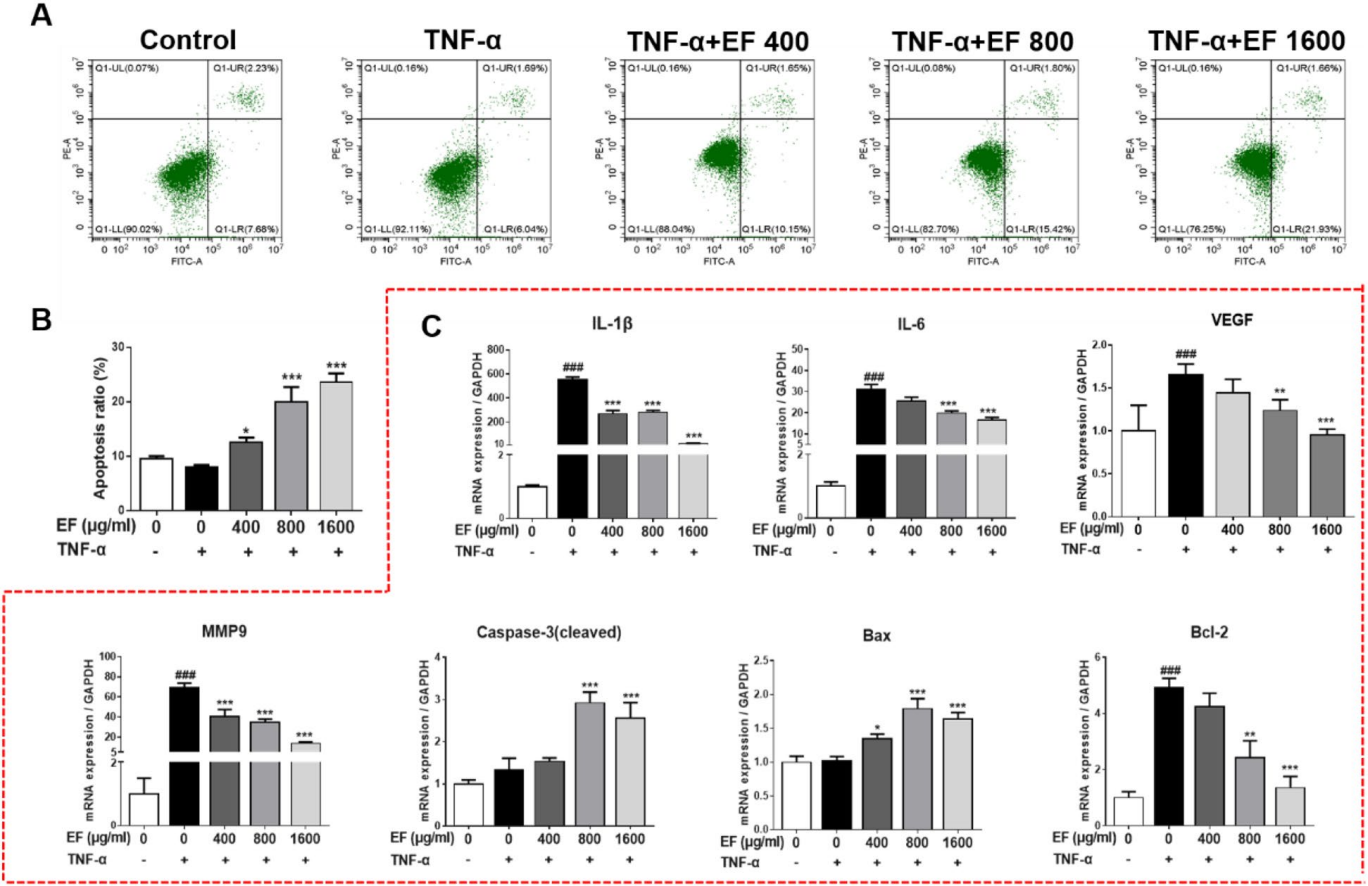
The apoptosis-inducing effects of EF in TNF-α-stimulated HFLS-RA cells. HFLS-RA cells were incubated with TNF-α (10 ng/mL) and EF (400, 800, 1600 µg/mL) for 24 h. **A** Percent apoptosis was determined by flow cytometry. Apoptotic cells were detected by staining with Annexin V-FITC/PI followed by flow cytometry. **B** Data are the mean ± SD (n = 4), *p < 0.05, **p < 0.01, ***p < 0.001, vs. TNF-α cells (HFLS-RA cells treated by TNF-α alone). ^#^p < 0.05, ^##^p < 0.01, ^###^>p < 0.001, vs. control cells. **C** mRNA expression of IL-1β, IL-6, VEGF, MMP-9, caspase-3, Bax, and Bcl-2 was determined by real-time RT-qPCR. Data are the mean ± SD (n = 4), *p < 0.05, **p < 0.01, ***p < 0.001, vs. TNF-α cells (HFLS-RA cells treated by TNF-α alone). ^#^p < 0.05, ^##^p < 0.01, ^###^p < 0.001, vs. control cells

The results shown above indicated that EF exhibited notable antiproliferative effects against HFLS-RA cells. We wished to ascertain the inhibitory effect of EF on the proliferation of HFLS-RA cells. Hence, we next investigated the ability of EF to induce the apoptosis of HFLS-RA cells by undertaking flow cytometry and RT-qPCR. There was a gradual increase in percent apoptosis as the concentration of EF increased (p < 0.05, p < 0.001) (Fig. 5A). mRNA expression of *IL-1β*, *IL-6*, *VEGF*, and matrix metalloproteinase (*MMP)-9* decreased significantly with increasing concentrations of EF (400, 800, 1600 µg/mL) when compared with that in the TNF-α group (p < 0.05, p < 0.01, p < 0.001) (Fig. 5B). Compared with the TNF-α group, mRNA expression of *cleaved caspase 3* and *Bax* in the EF group was significantly higher, and mRNA expression of *Bcl-2* in the EF group were significantly lower (p < 0.01, p < 0.001). These data indicated that EF could induce the apoptosis of HFLS-RA cells.

**Fig. 6:**
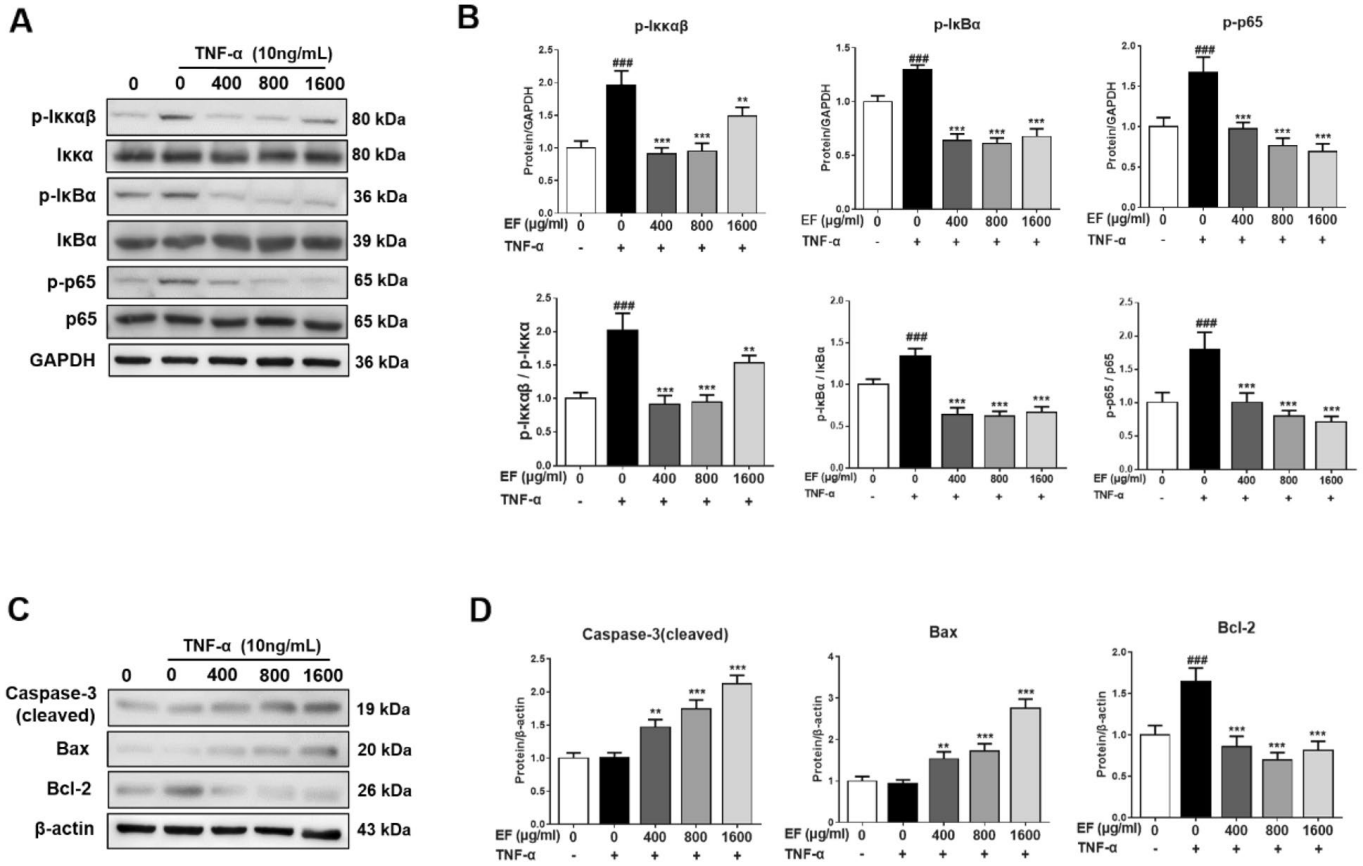
EF induced the apoptosis of TNF-α stimulated HFLS-RA cells *via* the NF-κB pathway. HFLS-RA cells were incubated with TNF-α (10 ng/mL) and EF (400, 800, 1600 µg/mL) for 24 h. **A**, **C** Western blotting was conducted to determine the protein expression of p-p65 NF-κB, p-Iκκαβ, p-IκBα, p65 NF-κB, Iκκαβ, IκBα, cleaved caspase3, Bax, and Bcl-2. Effect of EF on the surface expression of TNF-α receptors on HFLS-RA cells. **B**, **D** Data are the mean ± SD (n = 3), *p < 0.05, **p < 0.01, ***p < 0.001, vs. TNF-α cells (HFLS-RA cells treated by TNF-α alone). ^#^p < 0.05, ^##^p < 0.01, ^###^p < 0.001, vs. control cells

To further explore how EF induces the apoptosis of HFLS-RA cells, we measured the protein expression of factors related to the NF-κB pathway. Compared with the TNF-α group, the protein expression of p-Iκκ in the EF group (400, 800 µg/mL) was reduced significantly (p < 0.001) and the protein expression of p-IκB and p-p65 in the EF group (400, 800, 1600 µg/mL) was reduced significantly (p < 0.001) (Fig. 6A, B). In accordance with RT-qPCR results, the protein expression of cleaved caspase 3 and Bax in the EF group was significantly higher, whereas the mRNA expression of *Bcl-2* in the EF group was significantly lower (Fig. 6C, D). These results suggested that EF could induce the apoptosis of HFLS-RA cells via the NF-κB pathway.

### EF inhibited differentiation into osteoclasts in RANKL-stimulated RAW264.7 cells via the NF-κB pathway

**Fig. 7:**
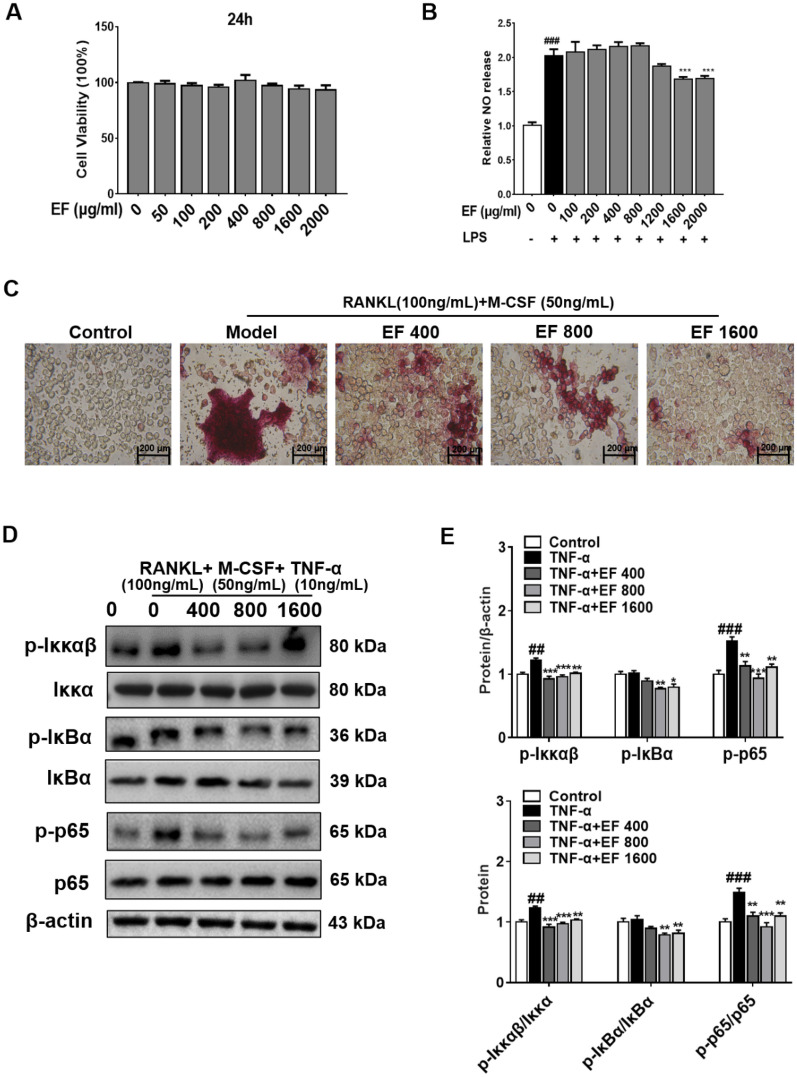
EF inhibited differentiation into osteoclasts in RANKL-stimulated RAW264.7 cells via the NF-κB pathway. **A** Toxicity of EF in cultured cells was tested for 24 h. **B** RAW264.7 cells were incubated with LPS (1 µg/mL) and EF (100, 200, 400, 800, 1600, 2000 µg/mL) for 24 h. **C** TRAP staining was used to investigate the effect of EF on the differentiation of RAW264.7 cells into osteoclasts. **D** Western blotting was conducted to determine the protein expression of p-p65 NF-κB, p-Iκκαβ, p-IκBα, p65 NF-κB, Iκκαβ, and IκBα. **E** Data are the mean ± SD (n = 3), *p < 0.05, **p < 0.01, ***p < 0.001, vs. TNF-α cells. ^#^p < 0.05, ^##^p < 0.01, ^###^p < 0.001, vs. control cells

The results shown above indicated that EF had a significant anti-proliferation effect upon HFLS-RA cells and could induce the apoptosis of HFLS-RA cells. We wished to investigate the effect of EF on the osteoclast differentiation of RAW264.7 cells. Hence, we investigated the effect of EF on the osteoclast differentiation of RAW264.7 cells induced by RANKL using TRAP staining and western blotting. The influence of EF on the viability of RAW264.7 cells was investigated with the CCK-8 assay. EF was not cytotoxic at 24 h (Fig. 7A). We investigated the effects of EF on the proliferation of lipopolysaccharide-stimulated RAW264.7 cells. EF (1600, 2000 µg/mL) could inhibit the proliferation of lipopolysaccharide-stimulated RAW264.7 cells (p < 0.01, p < 0.001) (Fig. 7B). Using TRAP staining and western blotting (Fig. 7C, D), we found that EF could inhibit the osteoclast differentiation of RAW264.7 cells induced by RANKL and inhibit activation of the NF-κB pathway (p < 0.05, p < 0.01, p < 0.001).

### EF exhibited significant anti-arthritis effects in a rat model of CIA

**Fig. 8:**
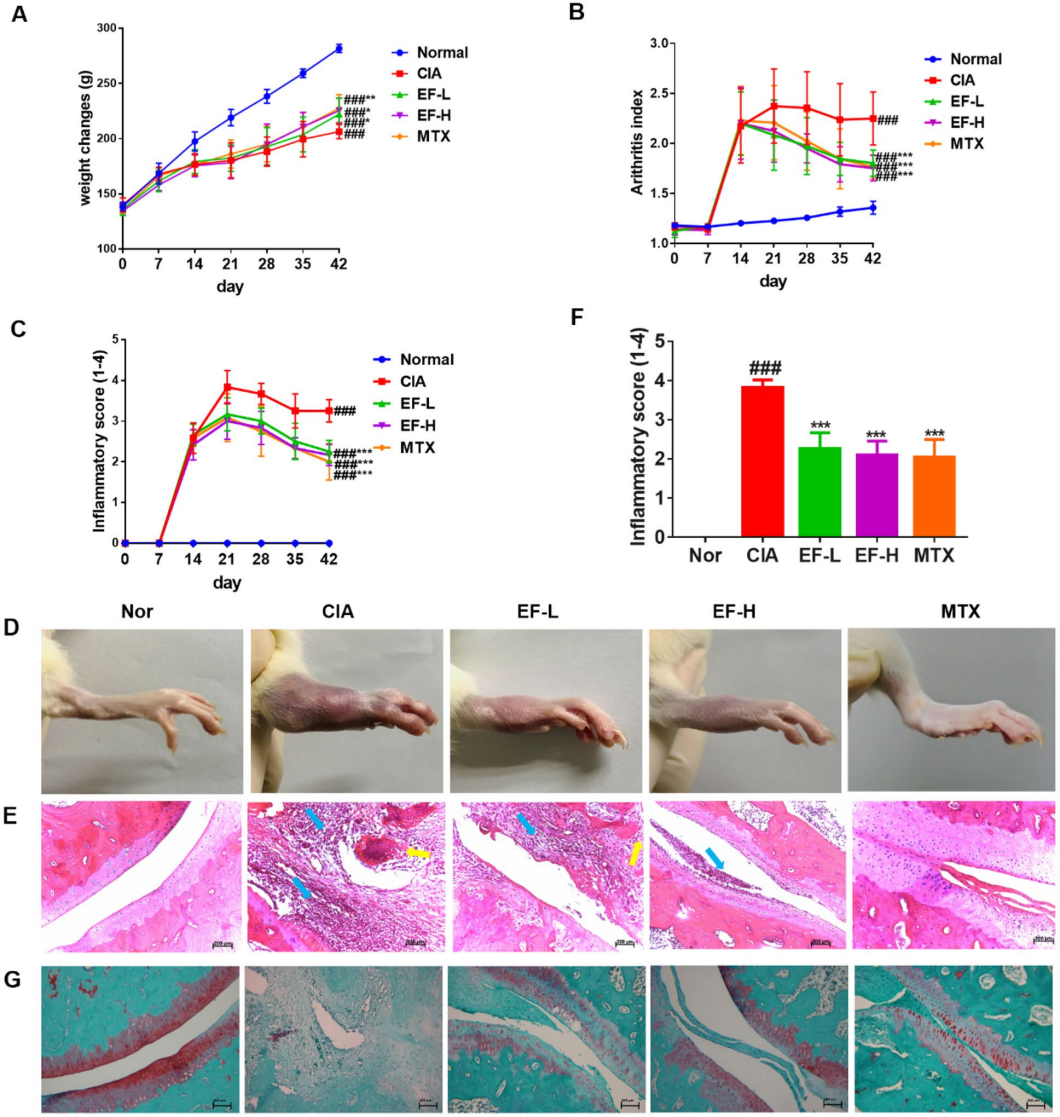
Anti-arthritis effects of EF on CIA rats. **A** Effects of EF on weight changes in CIA rats. **B**, **C** Effects of EF on paw volume and Arthritis Score changes in CIA rats. **D** A representative paw of rats in different treatment groups. **E**, **F** Tissue sections of ankle joints stained with hematoxylin and eosin (H&E). The blue arrow represents hyperplasia in the synovial membrane and pannus; the yellow arrow shows cartilage injury. **G** Tissue sections of ankle joints stained with Safranin O/Fast Green. Data are the mean ± SD (n = 6), *p < 0.05, **p < 0.01, ***p < 0.001, vs. CIA rats without treatment by EF or MTX, ^#^p < 0.05, ^##^p < 0.01, ^###^p < 0.001, vs. normal group

We wished to determine if EF could exhibit RA-based inflammatory effects leading to bone destruction in vivo, therefore we established a rat model of CIA. We initiated EF treatment 14 days after successful modeling. CIA rats weighed significantly less than rats in the control group; at day 21, the rats in the modeling group started to gain weight, but it was not until day 42 that significant differences in the weights of EF-L and EF-H groups were observed compared with the CIA group (p < 0.01) (Fig. 8A). CIA rats showed significant joint inflammation, and EF treatment reduced the severity of disease and joint swelling in CIA rats (p < 0.01, p < 0.001) (Fig. 8B–D). Histology revealed that the ankle joints of CIA rats exhibited infiltration of inflammatory cells in the soft tissue and lining of the synovial layer. Cartilage destruction led to pannus formation and narrowing of the joint space (Fig. 8E, F). EF treatment attenuated joint inflammation by reducing infiltration of inflammatory cells into the synovium and reducing pannus formation in CIA rats. Safranin O/Fast Green staining also showed that EF reduced cartilage damage in RA (Fig. 8G).

### EF effects on bone destruction

**Fig. 9:**
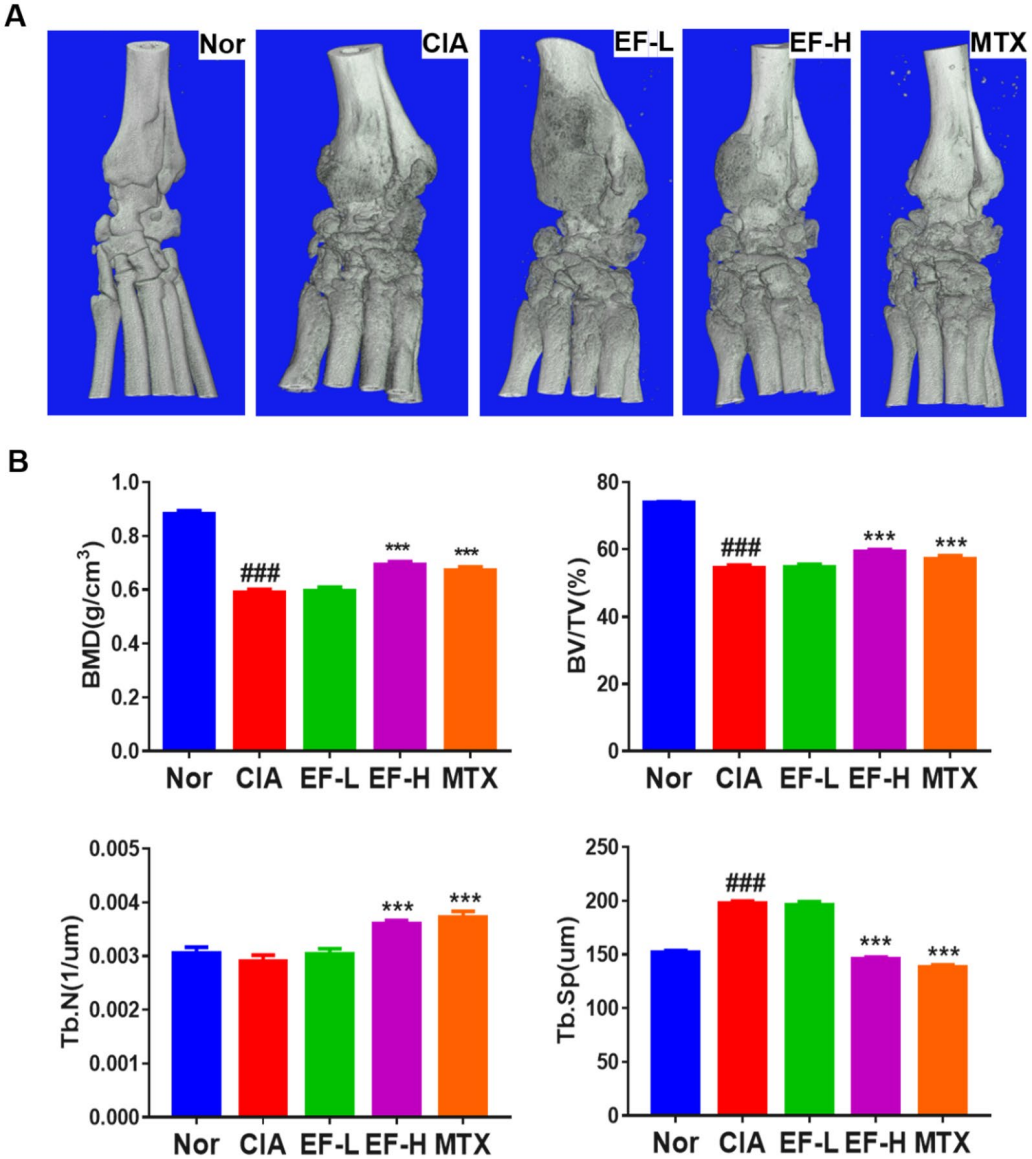
Analysis of micro-computed tomography (micro-CT) data. **A** All drugs were administered via the oral route, and methotrexate (MTX) was used as a positive control (1 mg/kg; administered once daily). EF was administered at 2 g kg^−1^ day^−1^ (EF-L) or 4 g kg^−1^ day^−1^(EF-H). **B** Analyses of bone-microstructure parameters: percentage BMD, BV/TV (%), Tb.N (µm), and Tb.Sp (µm). Data are the mean ± SD (n = 3), *p < 0.05, **p < 0.01, ***p < 0.001, vs. CIA rats without EF or MTX treatment, ^#^p < 0.05, ^##^p < 0.01, ^###^p < 0.001, vs. normal group

As EF treatment had a positive effect on pathologic bone destruction, we explored the potential of EF to reduce bone loss in rats. We undertook morphology experiments and created a 3D reconstruction of the tibia and ankle (Fig. 9A, B). We found that BMD and BV/TV were reduced significantly (p < 0.001). There was also a reduction in the quantity of bone trabecula, but this reduction was not significant. There was a significant increase in the separating degree of Tb.Sp (p < 0.001). In contrast, a high dose of EF prevented (at least in part) the sharp decline in bone mass and deterioration of trabecular microstructures in CIA rats. However, there were no significant differences when low-dose EF treatments were compared with controls.

### EF effect on the expression of bone-metabolism indices in serum and spleen

**Fig. 10:**
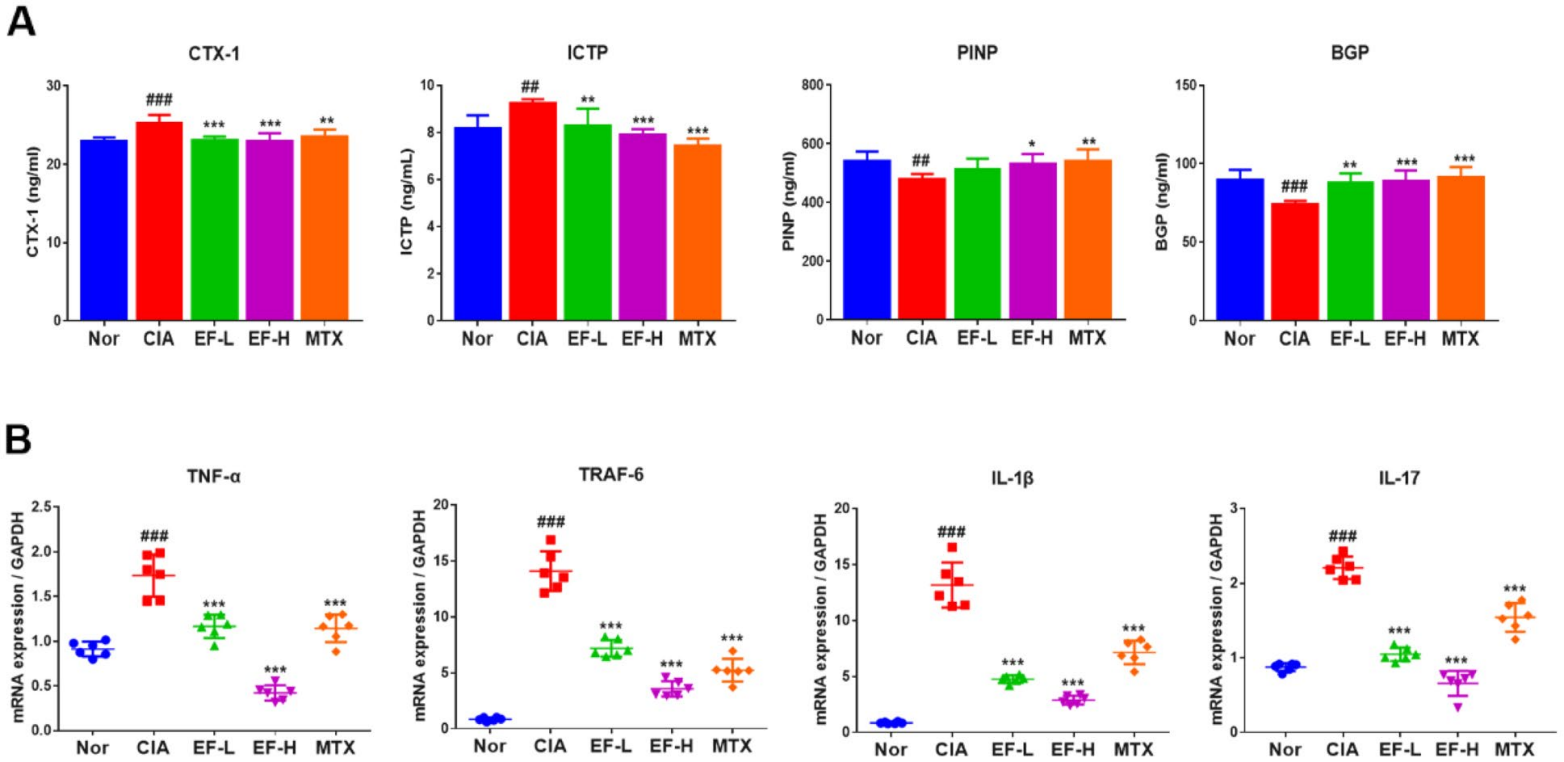
Levels of proinflammatory cytokines in the serum and spleen of rats as detected by ELISA and RT-qPCR. Drugs were administered via the oral route, and methotrexate (MTX) was used as a positive control (0.3 mg/kg, administered once daily). EF was administered at 2 g kg^−1^ day^−1^ (EF-L) and 4 g kg^−1^ day^−1^ (EF-H). **A** Effects of EF on the serum levels of CTX-1, ICTP, PINP, and BGP, in CIA rats. **B** Effects of EF on the levels of TNF-α, TRAF-6, IL-1β, and IL-17, in the serum of CIA rats. Data are the mean ± SD (n = 6), *p < 0.05, **p < 0.01, ***p < 0.001, vs. CIA rats without EF or MTX treatment, ^#^p < 0.05, ^##^p < 0.01, ^###^p < 0.001, vs. normal group

CTX-1, ICTP, PINP, and BGP are used commonly as clinical markers for osteoporosis detection. Increases and decreases in expression of these factors can reflect the status of bone destruction. Serum levels of CTX-1 and ICTP, and the mRNA expression of *TNF-α*, *TRAF-6*, *IL-1β*, and *IL-17*, in the spleen of rats in the CIA group were increased significantly compared with those in the control group. Serum levels of PINP and BGP in the CIA group were significantly lower in the CIA group than those in the control group (p < 0.05, p < 0.01, p < 0.001) (Fig. 10). Following EF administration, the levels of CTX-1 and ICTP, and mRNA expression of TNF-α, TRAF-6, IL-1β, and IL-17, in the spleen of rats were significantly lower than those in the CIA group; serum levels of PINP and BGP were also significantly higher than those in the control group (p < 0.05, p < 0.01, p < 0.001). EF treatment reduced the levels of proinflammatory factors in the spleen and regulated metabolic events in the bone; these observations were consistent with the micro-CT results and suggested that EF had a regulatory effect on the damage caused by osteoarthritis.

### EF effects on bone-metabolism indices in joint tissue

**Fig. 11:**
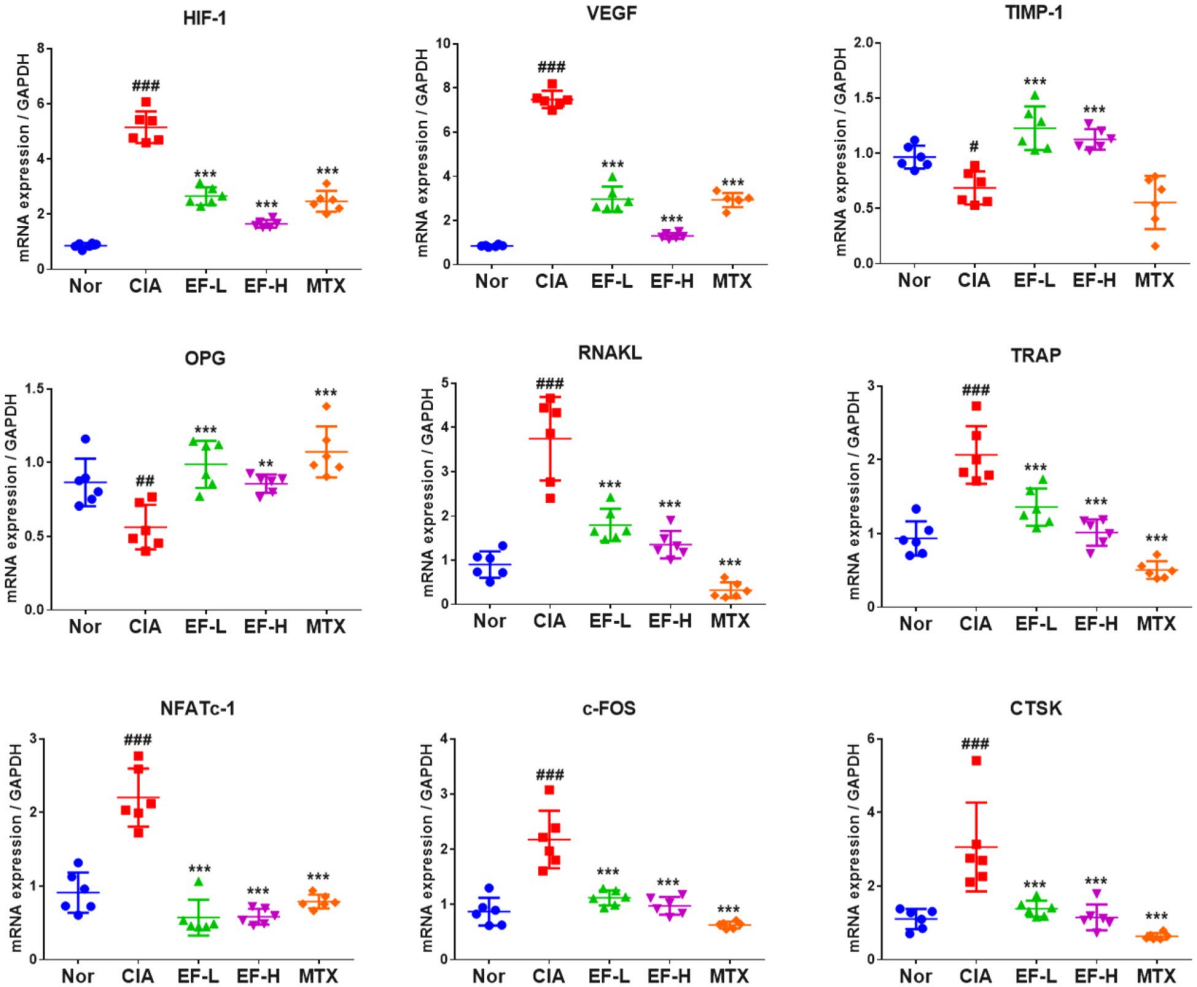
Expression of indicators of bone metabolism in rat cartilage tissue as determined by RT-qPCR. Drugs were administered via the oral route, and methotrexate (MTX) was used as a positive control (0.3 mg/kg, administered once daily). EF was administered at 2 g kg^−1^ day^−1^ (EF-L) and 4 g kg^−1^ day^−1^ (EF-H). Data are the mean ± SD (n = 6), *p < 0.05, **p < 0.01, ***p < 0.001, vs. CIA rats without treatment by EF or MTX, ^#^p < 0.05, ^##^p < 0.01, ^###^p < 0.001, vs. normal group

RANKL, c-FOS, nuclear factor of activated T cells (NFATc)1, cathepsin K (CTSK), and TRAP are considered to be markers for osteoclast production; VEGF and hypoxia inducible factor (HIF)-1 are considered to be related to angiogenesis. Increased levels of these factors (Fig. 11) indicated bone damage. Compared with the normal group, the levels of these factors were significantly higher in the CIA group, but significantly lower in the EF group than in the CIA group (p < 0.05, p < 0.01, p < 0.001). Tissue inhibitor of metalloproteinases (TIMP)-1 is the inhibitory factor for MMP-9 (a biomarker for the local inflammation of joints and associated with bone destruction). Levels of TIMP-1 and osteoprotegerin (OPG) in the EF group were increased significantly compared with those in the CIA group. Collectively, these data showed that EF could improve the osteoarthritic regulation of bone metabolism at the gene level.

### EF effects on the NF-κB pathway in joint tissue

**Fig. 12:**
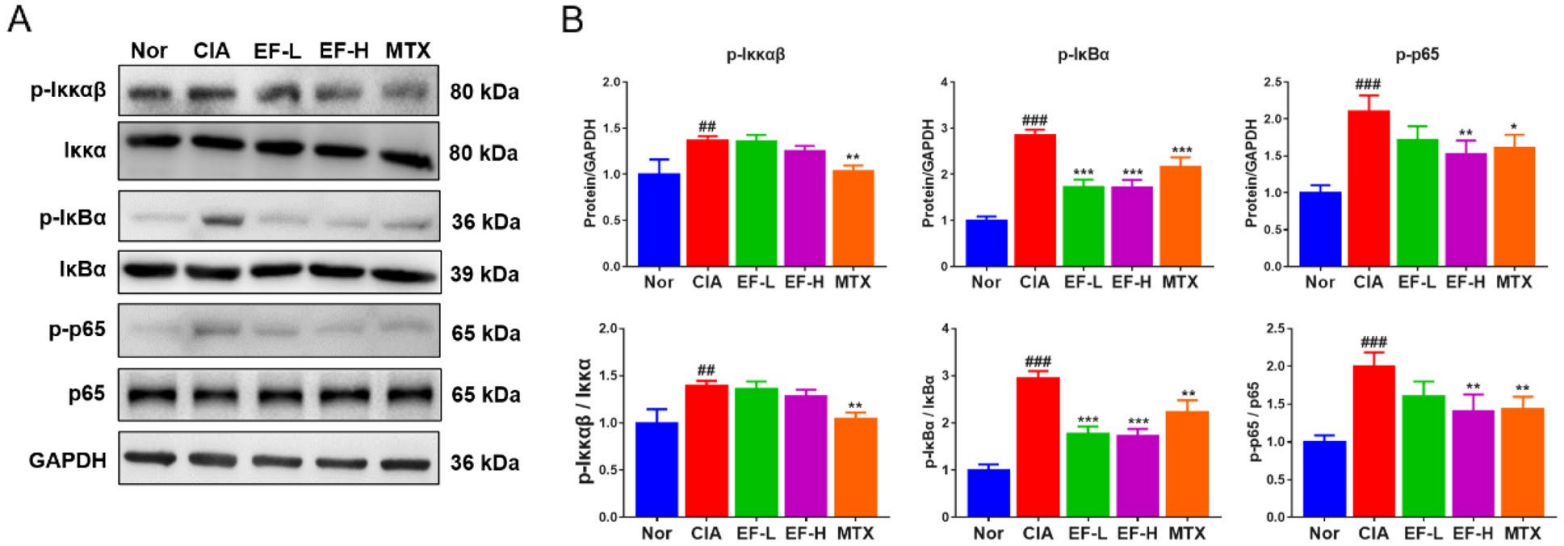
Western blotting was conducted to measure the expression of p-p65 NF-κB, p-Iκκαβ, p-IκBα, p65 NF-κB, Iκκαβ and IκBα. Drugs were administered via the oral route, and methotrexate (MTX) was used as a positive control (0.3 mg/kg, administered once daily). EF was administered at 2 g kg^−1^ day^−1^ (EF-L) and 4 g kg^−1^ day^−1^ (EF-H). Data are the mean ± SD (n = 3), *p < 0.05, **p < 0.01, ***p < 0.001, vs. CIA rats without treatment by EF or MTX, ^#^p < 0.05, ^##^p < 0.01, ^###^p < 0.001, vs. normal group

Next, we further explored the potential mechanism underlying the effects of EF on CIA rats (Fig. 12). We measured the protein expression of key factors in the NF-κB pathway. Compared with the CIA group, the protein expression of p-Iκκ, p-IκB, and p-p65 was significantly lower in the EF group (p < 0.05, p < 0.01, p < 0.001). These results suggested that EF could improve joint inflammation and bone destruction by inhibiting activation of the NF-κB pathway.

## Discussion

RA is a chronic and systemic autoimmune disease. RA pathogenesis is complex but the main pathologic features are synoviocyte proliferation and destruction of cartilage and bone. Collectively, these processes can cause a significant amount of pain and affect the quality of life adversely [12]. Joint symptoms can be divided into two types: synovial inflammation and destruction of joint structure. Once bone has started to undergo degeneration, it is very difficult to stop or reverse this process [1]. We showed that EF could significantly reduce the degree of joint swelling in a rat model of CIA. Pathologic sections further showed that EF could reduce partial necrosis of the articular cartilage, reduce pannus formation, changes in fibrous tissue, and synovial hyperplasia. Micro-CT showed significant improvement in bone destruction in the group of CIA rats treated with EF. These results suggest that EF can effectively inhibit the bone destruction experienced by joints in a rat model of CIA, and improve pathologic changes within joints.

Activated macrophages play an important part in the occurrence, maintenance, and pathogenesis of RA by secreting proinflammatory factors, including TNF-α. HFLS-RA cells are used commonly to investigate RA. If joint inflammation occurs, TNF-α levels increase significantly. Synoviocytes also proliferate and invade cartilage and bone, thereby leading to pannus formation. The excessive proliferation and inadequate apoptosis of FLS-RA cells is, in general, recognized as the pathologic basis of RA. There is increasing evidence to support the notion that regulating activation of the migration and invasion of HFLS-RA cells could prevent the destruction of RA joints [13]. It has also been reported that FLS in RA patients are resistant to apoptosis due to an imbalance of anti- and pro-apoptotic molecules (e.g., cleaved caspase 3, Bax) and that anti-apoptotic mediators may have a key role, including Bcl-2 [14]. In addition, HFLS-RA cells show highly proliferative behavior similar to that of tumor cells, therefore we conducted proliferation experiments [15]. We used TNF-α to stimulate HFLS-RA cells. EF inhibited the proliferation of HFLS-RA cells in colony-based and EdU-based experiments. EF also promoted the apoptosis of HFLS-RA cells as determined by flow cytometry. RT-qPCR and western blotting further demonstrated that protein expression of p-p65 NF-κB, p-Iκκαβ, and p-IκBα was reduced significantly upon EF treatment. Increased levels of caspase 3 and Bax, and decreased levels of Bcl-2, demonstrated that EF could promote apoptosis by inhibiting activation of the NF-κB pathway. In addition, EF could significantly inhibit NO release in lipopolysaccharide-induced RAW264.7 cells without causing cytotoxicity. Therefore, we further induced RAW264.7 cells with RANKL and M-CSF, and found that EF could significantly inhibit the differentiation of RAW264.7 cells into osteoclasts, and inhibit activation of the NF-κB pathway in osteoclasts. Therefore, we speculated that the effect of EF on the improvement of bone destruction may be related to inhibition of activation of the NF-κB pathway and osteoclast generation.

FLS-RA cells cause an increase in MMP expression, increase the rate of degradation in the extracellular matrix of cartilage, increase the migration and invasion of cartilage and bone, and cause infiltration of inflammatory cells and joint destruction [15]. Some studies have found that the intensification of bone resorption is a major factor of joint destruction in RA, and that an increase in the CTX-1 level in serum is closely related to bone resorption [16]. Therefore, several factors related to bone resorption and bone formation were measured in our study: CTX-1, ICTP, PINP, and BGP. We found that EF treatment reduced the levels of CTX-1 and ICTP, but increased the levels of PINP and BGP, in rat serum. TNF-α, IL-1, and IL-6 stimulate neutrophil migration, promote osteoclast maturation, and promote VEGF secretion, thereby leading to joint destruction [17]. In the present study, EF reduced the levels of several factors in the spleen of CIA rats: TNF-α, TRAF-6, IL-1β, and IL-17. Also, the expression of VEGF and HIF-1, along with expression of angiogenesis-related factors in the cartilage of rats, were reduced significantly in pathologic tissue sections.

The NF-κB pathway is associated with angiogenesis in RA. The increase in VEGF expression induced by RANKL is mediated by NF-κB in osteoclasts [17]. Osteoclasts are the only cells capable of absorbing bone in the body. Bone resorption is achieved by close interaction with extracellular-matrix proteins. In RA, RANKL activates NF-κB signaling, as well as matrix enzymes that are synthesized by osteoclasts (CTSK, c-FOS, TRAP). Collectively, these factors degrade the bone matrix and exacerbate bone erosion [18]. RANKL signaling acts *via* its receptor activator, RANK, and has an important osteoclastic role in bone remodeling [19]. In the CIA group of rats, we observed a significant increase in the levels of osteoclast markers (TRAP, NFATc1, CTSK, c-FOS, RANKL), which indicated that CIA rats had suffered significant levels of bone destruction; collectively, these factors had induced severe corrosion in cartilage and bone tissue. Following administration, EF significantly reduced the mRNA expression of *TRAP*, *NFATc1*, *CTSK*, *c-FOS*, and *RANKL*. The mRNA expression of OPG (a marker of osteogenesis) was significantly higher in the EF group (though this showed some variation). The MMP family is involved in tissue remodeling, tissue repair, and angiogenesis, and is regulated by TIMP. In osteoarthritis or RA, the expression of the family of MMP proteins is upregulated, and the balance with TIMP is destroyed [20]. We observed reduced levels of TIMP1 in the cartilage of rats in the model group. In contrast, levels of TIMP1 in the EF group increased to varying degrees, which indicated that EF could improve arthritis. However, whether EF influences expression of MMP proteins requires further study.

Previously, we preliminarily discussed the ameliorative effects of EB, EL, and EF on inflammation in a rat model of CIA (hereafter termed “CIA rats”) [11]. In the early stage, we also preliminarily explored 27 components contained in EF [21]. We hypothesized that EF has the same effect as EB, which may be related to the similarity of their components.

Here, based on previous research, we further investigated the effect of EF on: (i) the proliferation, migration, and apoptosis of human fibroblast-like synoviocytes in rheumatoid arthritis (HFLS-RA); (ii) osteoclast differentiation in RAW264.7 cells; (iii) bone destruction via the NF-κB pathway in a rat model of CIA.

We provided an experimental basis for EF application, but our study had a limitation based on its design: we could not clarify the influence of an active single component of EF and its effect on HFLS-RA cells. This information gap will be studied in the future by our research team. In summary, the expression of p-p65 NF-κB, p-Iκκαβ, and p-IκBα in the cartilage of model-group rats was upregulated significantly compared with that in the control group. After treatment, the expression of p-p65 NF-κB, p-Iκκαβ, and p-IκBα in the EF group was reduced significantly. These data demonstrated that EF may improve bone destruction in a rat model of CIA by inhibiting the NF-κB pathway.

## Conclusions

EF hampered proliferation of HFLS-RA cells, promoted apoptosis, and hindered osteoclast differentiation by inhibiting activation of the NF-κB pathway. EF also reduced toe swelling in a rat model of CIA, inhibited the expression of pro-angiogenic factors, and delayed the destruction of articular cartilage and bone. The mechanism underlying the effects of EF was related to bone metabolism induced by the NF-κB pathway. The present study will help to elucidate the precise pharmacologic mechanisms underlying the actions of EF, and provide an experimental basis for the application of EF in the clinical treatment of RA.

## Data Availability

The datasets used and/or analyzed during the current study are available from the corresponding author upon reasonable request.

## Abbreviations

Bax: Bcl-2-associated X protein
Bcl-2: B-cell lymphoma-2
BGP: Bone galprotein
caspase 3: Cysteinyl aspartate specific proteinase-3
CTSK: Cathepsin K
CTX-1: C-terminal telopeptide I
EF: The male flower of *Eucommia ulmoides* Oliv.
HIF-1: Hypoxia inducible factor-1
ICTP: Cross-linked carboxy-terminal telopeptide of type I collagen
IL-17: Interleukin-17
IL-1β: Interleukin-1β
IL-6: Interleukin-6
LPS: Lipopolysaccharide
MMP-9: Matrix metalloprotein-9
NFATc-1: Nuclear factor of activated T cells-1
OPG: Osteoprotegerin
PINP: Propeptide of type I procollagen
RA: Rheumatoid arthritis
RANKL: Receptor activator of nuclear factor-kappa B ligand
TIMP-1: Tissue inhibitor of metalloproteinases-1
TRAF-6: Tumor necrosis factor receptor-associated factor-6
TRAP: Tartrate resistant acid phosphatase
TNF-α: Tumor necrosis factor
VEGF: Vascular endothelial growth factor
